# Overall Survival in Patients with Stage IV Pan-NET Eligible for Liver Transplantation

**DOI:** 10.1007/s00268-022-06736-1

**Published:** 2022-09-29

**Authors:** Josefine Kjaer, Sara Smith, Per Hellman, Peter Stålberg, Joakim Crona, Staffan Welin, Olov Norlén

**Affiliations:** 1 Department of Surgical Sciences, Uppsala University, 751 85 Uppsala, Sweden; 2 Department of Medical Sciences, Uppsala University, 751 85 Uppsala, Sweden

## Abstract

**Background:**

The use of liver transplantation (LT) in patients with stage IV neuroendocrine pancreatic tumors (pan-NET) is under debate. Previous studies report a 5-year survival of 27–53% after LT in pan-NET and up to 92.7% in patients with mixed NETs. This study aimed to determine survival rates of patients with stage IV pan-NET meeting criteria for LT while only subjected to multimodal treatment.

**Methods:**

Medical records of patients with pan-NET diagnosed from 2000 to 2021 at a tertiary referral center were evaluated for eligibility. Patients without liver metastases, who did not undergo primary tumor surgery, age > 75 years and with grade 3 tumors were excluded. The patients were divided into groups; all included patients, patients meeting the Milan, the United Network for Organ Sharing (UNOS) or the European Neuroendocrine Tumor Society (ENETS) criteria for LT. Kaplan–Meier survival analysis was used to calculate overall survival.

**Results:**

Out of 519 patients with pan-NET, 41 patients were included. Mean follow-up time was 5.4 years. Overall survival was 9.3 years (95% Cl 6.8–11.7), and 5-year survival was 64.7% (95% CI 48.2–81.2). Patients meeting the Milan, ENETS and UNOS criteria for LT had a 5-year survival of 64.9% (95% CI 32.2–97.6), 85.7% (95% CI 59.8–100.0) and 55.4% (95% CI 26.0–84.8), respectively.

**Conclusions:**

In patients with stage IV pan-NET, grade 1 and 2, with no extra abdominal disease, 5-year survival was 64.7% (95% CI 48.2–81.2). As these survival rates exceed previously published series of LT for pan-NET, the evidence base for this treatment is very weak.

## Introduction

Pancreatic neuroendocrine tumors (pan-NET) are a heterogeneous group of tumors, arising from the endocrine cells of the pancreas, with an increasing incidence [1, 2]. Non-functioning pan-NET are the most common and are often diagnosed at stage IV due to the absence of hormonal symptoms [3]. More than 50% of patients either present with, or develop, liver metastases (LM) [4, 5]. According to data from the Surveillance, Epidemiology and End Results (SEER) database, median survival for pan-NET with distant metastases is 25 months, compared to 230 months and 7,5 years for localized and regional disease, respectively [1]. Overall, the reported 5-year survival rate for stage IV pan-NET is 25–60% [6, 7].

The only curative treatment for pan-NET is complete surgical resection, regardless of tumor stage or grade. Even if hepatic surgery is the only treatment offering potential cure for NET liver metastasis, it is rarely achieved even with complete elimination of the hepatic tumor burden [8]. This is probably due to the high risk of remaining liver-micrometastases after resection that has been proven in 32% of patients subjected to liver resection, suggesting that even with attempted curative resection, cure is difficult to achieve [9]. In the setting of unresectable metastatic disease, other treatment options include trans-arterial chemoembolization, radiofrequency or microwave ablation, peptide receptor radionuclide therapy (PRRT), systemic chemotherapy and molecular-targeted therapies [10–12]. In patients with complete resection of the primary tumor and loco-regional lymph node metastases, liver transplantation (LT) has also been suggested as a therapeutic method in an attempt to prolong survival rates [13].

The selection criteria for LT vary among transplant centers and countries. The most common used criteria for LT are the Milan criteria, LT criteria according to the European Neuroendocrine Tumor Society (ENETS) guidelines and LT criteria according to the United Network for Organ Sharing (UNOS) guidelines [12, 14–16]. Most of the studies presenting survival rates after LT for neuroendocrine tumors include NET tumors of different origin. The largest study to date is a multicenter study of 213 patients with mixed NET, where the 5-year overall survival (OS) was 52%. A sub-analysis of the 94 patients with pan-NET and 3 patients with duodenal NET showed 5-year OS of 44% [17]. In total, series that report survival explicitly for pan-NET, the 5-year survival ranges between 29 and 53% [18–21]. In metastatic NET of unspecified origin, survival rates after LT vary from 47 to 97.2% [18, 22–26].

Currently, there are no globally accepted selection criteria for LT in patients with Pan-NET.

North America Neuroendocrine Tumor Society (NANETS) guidelines do not include LT as a treatment for Pan-NET; however, for small intestine neuroendocrine tumors (SI-NET), it may be an option for some patients [27].

### Aim of study

The aim of this study is to determine the survival in a cohort of patients with stage IV Pan-NET that meet the Milan criteria, ENETS guidelines and UNOS guidelines for LT, but were only given multimodal treatment.

## Materials and method

### Study design and study population

A retrospective cohort study was conducted. Medical records of patients with pan-NET diagnosed from 2000 to 2021 and treated at the Uppsala University Hospital in Uppsala, Sweden, were screened for inclusion. Patients without liver metastases, patients who did not undergo primary tumor surgery, age > 75 years, patients with grade 3 tumors and extra abdominal disease were excluded. Also, due to lack of follow-up, all patients with non-Swedish personal numbers were excluded. To ensure the quality of data reporting, the STROBE statement was followed [28].

### Patient data

Variables chosen for baseline patient characteristics were age, gender, genetic syndrome, functioning or non-functioning tumor, hormonal syndrome, Charlson Comorbidity Index (CCI), total s-bilirubin and albumin levels. Tumor-related characteristics included WHO grade, Ki-67 index and liver metastases load. The different hormonal syndromes were determined by clinical symptoms in concordance with hormonal levels. Comorbidity was assessed according to the Charlson Comorbidity Index (CCI), a validated prognostic indicator for mortality in various disease subgroups [29, 30]. Tumor proliferation using Ki-67 index was determined at the pathology department, and the patients were graded according to the WHO 2017 classification. If Ki-67 index was assessed in the liver metastases as well, and the grade was discrepant with a higher grade in the metastasis, the metastatic indices were used. Patients with neither Ki-67 index nor mitotic rate reported were classified as unknown.

Dates of diagnosis, primary tumor surgery, systemic treatment, treatment of liver metastases, last date of follow-up and date of death were noted. To avoid immortal time bias, a time zero was defined. For patients that met criteria for LT according to any of the criteria above, time zero was defined as the date when the patient was eligible for LT and for patients that did not meet the criteria for LT, time zero was set when the following criteria were met; first appointment at Uppsala University Hospital, primary tumor resected and LM present. Survival was calculated from time 0 to time of death or censured at last follow-up. Progressive disease was defined by comparing two sequent CT scans within 6 months from time 0 and assessed according to the RECIST 1.1 criteria. Progression was defined as liver metastases growth > 20% or occurrence of new metastases [31]. Presence of lymph node metastases was determined by either uptake on Ga-68-dotatoc-PET scan, size > 1 cm and suspect metastatic characteristics on a CT-scan or pathology-verified. The lymph node metastases were further defined as regional or distant based on the Japanese Pancreas Society (JPS) classification [32]. Distant lymph node metastases were defined as extrahepatic disease.

All patients included in the study were thereafter investigated according to the above-mentioned criteria and guidelines and then sorted into the following groups; all patients meeting the inclusion criteria, Milan-NET criteria met, UNOS criteria met and ENETS criteria met.

### Milan criteria

The Milan criteria were developed in 1995 at the National Cancer Institute of Milan [15]. The five absolute criteria developed were histologic grade G1 or G2, portal drainage of the primary tumor, pre-transplant curative resection of all extrahepatic lesions, stable disease > 6 months and hepatic tumor burden < 50%. Age is a relative criteria, in our study < 65.

### ENETS guidelines

Criteria for LT according to the European Neuroendocrine Tumor Society (ENETS) guidelines from 2012 are patients with well-differentiated pan-NET with Ki67 index < 10%, primary tumor removed at least 6 months before transplantation, < 50% liver involvement or < 75% liver involvement in patients with refractory hormonal symptoms, stable disease for > 6 months, age < 55 and diffuse unresectable disease confined to the liver with no extrahepatic disease [14]. An update from 2016 suggests patients with well-differentiated pan-NET, no extrahepatic disease and low serum total bilirubin. Young age and functional syndromes that are early refractory to all other treatment options are preferable [12].

### UNOS guidelines

The United Network for Organ Sharing (UNOS) guidelines are mostly based on the Milan criteria; NET G1 or G2, resection of primary tumor and extrahepatic lesions without recurrence > 6 months, total liver volume < 50% and age < 60 years. Additional criteria include unresectable LM, negative metastatic workup by PET-scan, lack of extrahepatic tumor recurrence last 6 months and radiographic characteristics of neuroendocrine LM. If lymph node metastases are found by PET-scan, a follow-up scan after 3 months should be negative before re-listing [16]. In this study, we added 3-month follow-up and if stable disease (no size progression of the lymph node metastasis or new metastasis) we assessed the patients as eligible for the study.

### Outcomes/Endpoints

Primary endpoint was overall 5-year survival in percent [33]. As all patients had metastatic disease, cause of death was assumed to be pan-NET, if no other obvious cause was reported in the patient´s medical records.

### Data analysis and statistic method

Statistical analyses were calculated with SPSS (IBM Corp, Armonk, NY). Kaplan–Meier analysis was used to compute overall survival, and log-rank test was performed to compare survival between groups.

### Ethics

The Swedish Ethical Review Board approved the study (no. 2012/160 and 2020–05,645). Personal data were processed in line with the General Data Protection Regulation (GDPR).

## Results and discussion

**Fig. 1:**
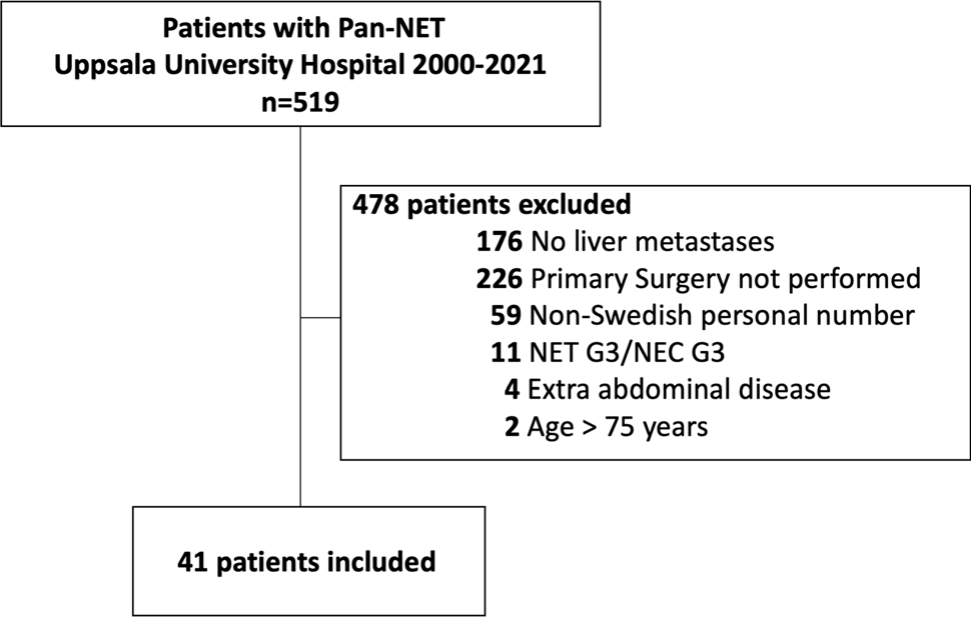
Flow-chart of patients enrolled in the study

In total, 519 patients were diagnosed with Pan-NET at Uppsala University Hospital from 2000 to 2021 and screened for inclusion in our study. Of these, patients were excluded due to absence of liver metastases (*n* = 176), no primary tumor resection (*n* = 228), non-Swedish personal number, NET/NEC G3 tumors (*n* = 11), patients with age > 75 years (*n* = 2) and extra abdominal disease (*n* = 4). After exclusion, 41 patients remained. (Fig. 1) Thereafter, these patients were investigated for meeting the criteria for LT according to the different guidelines. In total, 25 of the 41 included patients did not meet the criteria for LT according to the above-mentioned criteria and guidelines. Twelve patients did not have stable disease for 6 months at any point, 10 patients were over 65 years old at the timepoint of stable disease and three patients were both > 65 and did not have stable disease. Two patients had suspicious small regional lymph node metastases, in both case stable according to radiology, and they could therefore be included in the UNOS group according to present criteria.

### Baseline characteristics

Of the included 41 patients, 51.2% were women, 26.8% had a functioning tumor and 12.2% had a genetic syndrome. The majority of patients, 65.9%, had a NET G2 and 24.4% of the patients had ten or more liver metastases. (Table 1).

**Table 1:** Baseline characteristics for patients in each group

Baseline characteristics	Patient groups			
	Patients meeting inclusion criteria	Milan criteria	ENETS guidelines	UNOS guidelines
Nr of patients	41	11	8	13
*Age (years)*				
< 55 55–65 66–75	17 (27.0) 13 (44.8) 11 (50)	9 (81.8) 2 (18.2) 0	8 (100) 0 0	12 (92.3) 1 (7.7) 0
*Sex*				
Female	21 (51.2)	6 (54.5)	6 (75)	8 (61.5)
Hormonal expression Functioning	11 (26.8)	2 (18.2)	3 (37.5)	5 (38.5)
*Genetic Syndrome*				
MEN-1 VHL	4 (9.8) 1 (2.4)	1 (9.1) 0 (0)	0 (0) 0 (0)	2 (15.4) 0 (0)
*CCI* 0 1 2 > = 3	21 (51.2) 13 (31.7) 5 (12.2) 2 (4.9)	8 (70.6) 2 (18.2) 1 (9.1) 0	5 (62.5) 2 (25) 1 (12.5) 0	10 (76.9) 2 (15.4) 1 (7.7) 0
*WHO Grade*				
NET G1 NET G2	14 (34.1) 27 (65.9)	4 (36.4) 7 (63.6)	3 (37.5) 5 (62.5)	3 (23.1) 10 (76.9)
*Number of LM*				
1–3 4–9 > 10	19 (46.3) 12 (29.3) 10 (24.4)	2 (18.2) 5 (45.5) 4 (36.4)	4 (50) 1 (12.5) 3 (37.5)	5 (38.5) 3 (23.1) 5 (38.5)
*Size of largest LM (cm)*				
	1.5 (1.0–2.0) ^#^	1.0 (0.9–3.0) ^#^	1.0 (0.9–1.8) ^#^	1.5 (1.0–2.2) ^#^
*Time period (Time 0)*				
2000–2010 2011–2021	18 (43.9) 23 (56.1)	7 (63.6) 4 (36.3)	4 (50.0) 4 (50.0)	7 (53.8) 6 (46.2)
Time from diagnosis to Time 0	0.9 (0.3–3.3) ^#^	3.0 (0.9–5.5) ^#^	1.6 (0.5–4.8) ^#^	3.0 (0.9–5.6) ^#^

Numbers in parenthesis is percentages if not stated otherwise; #Median (IQR)

*CCI* Charlson Comorbidity Index, *LM* liver metastases

Four groups were constituted; all patients that met the inclusion criteria in our study (*n* = 41), patients meeting the Milan criteria for LT met (*n* = 11), the criteria for LT according to ENETS guidelines met (*n* = 8) and the criteria for LT according to UNOS guidelines met (*n* = 13). In total, 16 patients were included in one or more of the LT-groups.

Patients received medical treatment after individual assessment according to current ENETS guidelines. All patients but one, received first line medical treatment and 75.6% received second line medical treatment. Many patients were also given liver-specific multimodal treatment with hepatic resection (12.2%), thermal hepatic ablation (36.6%) and liver embolization (14.6%) (Table 2). The medical treatment is specified in Table 3.

**Table 2:** Multimodal treatment received for patients in each group

Treatment	Patient groups			
	Patients meeting inclusion criteria	Milan criteria	ENETS guidelines	UNOS guidelines
Nr of patients	41	11	8	13
First line medical treatment	40 (97.6)	11 (100)	8 (100)	13 (100)
Second line medical treatment	32 (78.0)	10 (90.4)	6 (75.0)	12 (92.3)
Third line medical treatment	15 (36.6)	5 (45.5)	3 (37.5)	7 (53.8)
Hepatic resection	5 (12.2)	2 (18.2)	1 (12.5)	3 (23.1)
Hepatic Ablation	15 (36.6)	5 (45.5)	8 (25.0)	7 (53.8)
Liver embolization	6 (14.6)	1 (9.1)	0	1 /7.7)

Numbers in parenthesis is percentages

**Table 3:** Medical treatment for all patients included in the study

Medical treatments	First line (*n* = 40)	Second line (*n* = 32)	Third line (*n* = 15)
Somatostatin analogues	18	2	1
Streptotocin + Fluorouracil	20	6	3
Carboplatin	1	2	
Cisplatin		1	
Temezolomid		9	4
PRRT		8	5
Everolimus		1	1
Interferon	1	3	1

The numbers represent numbers of patients given the treatment

### Overall survival

Overall survival for all patients was 9.3 years (95% Cl 6.8 to 11.7), and 5-year survival was 64.7% (95% CI 48.2–81.2). Mean follow-up time was 5.4 years. Number of events at study endpoint was 23.

**Fig. 2:**
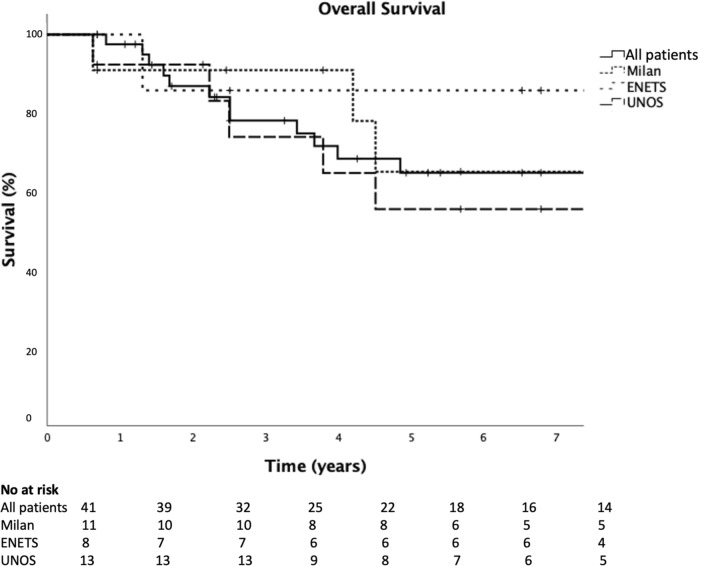
Kaplan–Meier survival analyses for all groups

Patients that med the Milan criteria for LT had a 5-year survival of 64.9% (95% CI 32.2–97.6), for the patients that met the ENETS guidelines for LT 85.7% (95% CI 59.8–100.0) and 55.4% (95% CI 26.0–84.8) for the patients that met the UNOS guidelines for LT (Fig. 2).

When dividing the patients according to time period, 2000–2010 and 2011–2021 5-year survival from time zero was 55.6% (95% CI 32.6–57.9) and 76.3% (55.5–97.1) log-rank *p* = 0.378.

Patients with stage IV pan-NET, grade 1 and 2, with no extra abdominal disease undergoing multimodal treatment have a 5-year survival of 64.7% (95% CI 48.2–81.2). This is comparable or even higher than those survival rates reported in patients undergoing LT. The patients that met the Milan criteria for LT had a 5-year survival of 64.9% (95% CI 32.2–97.6), patients that met the criteria according to the ENETS guidelines 85.7% (95% CI 59.8–100.0) and patients that met the criteria according to the UNOS guidelines 55.4% (95% CI 26.0–84.8), which all is higher than the reported 27–53% 5-year survival after LT for pan-NET disease [17–21]. Most of the studies reporting long-term outcome after LT in patients with NET include NET of mixed origin and only few specified the survival rates according to primary tumor origin [17–20]. When SI-NET is analyzed together with pan-NET as gastrointestinal enteropancreatic neuroendocrine tumors (GEP-NET) regarding survival, the most likely result is an overestimate of the expected survival after LT for pan-NET, as SI-NET is both more common and has a better prognosis [34]. In a study by Mazzafero et al., a very high survival rate after LT for GEP-NET is reported; the transplanted cohort had a remarkable 5-year survival of 97.2% and 10-year survival of 88.8% [26]. However, in comparison to the present study, the study included various NETs and therefore the total survival rates are expected to be higher than for pan-NET. In the same study by Mazzafero et al., 88 patients out of 280 were eligible for LT but only 42 patients underwent LT and only 15 of those were patients with pan-NET. Of note, those undergoing LT had notably lower TNM-stage and tumor grade, were younger, received locoregional therapies and less somatostatin analog therapy than the group who did not receive a transplant but were eligible, and these factors also probably influence the prognosis of the transplanted group. Apart from the above-mentioned inherent selection bias for LT in the study by Mazzafero et al., immortal time bias was also evident; the survival was measured from surgery of the primary tumor and not from the transplantation date, a median time of 18.5 months. This will favor the LT group in terms of overestimating the survival and thereto make 30- or 90-day mortality after baseline non-existent in their analysis as most patients in the transplant group are still on the waitlist at this timepoint.

In the current study, only 41 of the 519 patients fulfilled the general inclusion criteria and only 16 (3.1%) patients met the criteria for LT according to one or more of the three guidelines analyzed. The low rate of patients eligible for LT makes the clinical implication of LT in pan-NET disease debatable, and the relatively high survival in patients eligible for LT according to current criteria raises concern about the use of LT for NET outside clinical trials. This is even more evident when factoring in the postoperative mortality of 5–14% after LT, a clear risk of postoperative complications and compulsory life-long immunosuppressive drugs [17, 25]. Other concerns regarding LT in general are post-transplant malignancy due to the immunosuppressive treatment which includes both recurrence of the original cancer or development of other cancers. However, recurrence rates after LT in various NET vary and have been reported between 31.3 and 40.3% with a median time of 17–52 months [17, 24]. With all of the above in mind, and similar survival rates with multimodal treatment, LT is most likely not beneficial to most pan-NET patients. On the other hand, it needs to be acknowledged that the lack of a non-biased treatment group to compare the current data with and the narrow selection criteria for LT may have caused us to overlook patients that indeed would benefit from LT.

Another aspect is health-related quality of life (HRQoL), which in general after LT seems to be fairly comparable with age-matched groups [35]. None of the above studies have evaluated HRQoL, and as patients with remaining liver metastases may suffer from hormonal symptoms, pressure symptoms or side effects of multimodal treatment, it would be interesting to further investigate the HRQoL in pan-NET patients after LT.

### Limitations

As with all retrospective studies, there may be limitations in interpreting data from medical records that could influence the results. In this study, the ENETS guidelines for LT from 2012 were used as the criteria are more clearly stated than the updated 2016 version. If the updated ENETS guidelines from 2016 had been used instead, which also state that young age and functional pan-NET are preferable, only one patient would have met the criteria.

Due to the rareness of pan-NET disease, and the narrow selection criteria for LT in GEP-NET disease, the number of patients eligible for inclusion in this study resulted in a relatively small sample size. The low number of patients makes any conclusions difficult to draw and the level of evidence of this study is weak, in fact about as weak as contemporary case series of transplanted pan-NET patients propagating for liver transplant, as these also included an equal low number of patients with pan-NET [17–20].

As with all medical (and surgical treatments), the burden of the proof (evidence) needs to be on the doctor/surgeon that wants to provide a novel treatment, not on the doctor that treats patients according to clinical routine. This is especially true if there is a risk of adverse events with the novel treatment, which is certainly the case regarding liver transplantation, with some multicenter series stating 10–14% 90-day mortality post LT [17, 19].

To conclude, this retrospective cohort study focused on patients with stage IV pan-NET eligible for LT with grade 1 and 2 tumors, no extra abdominal disease, undergoing multimodal treatment, and benchmarks the 5-year survival to 64.7% (95% CI 48.2–81.2). As these survival rates exceed previously published series of LT for pan-NET, the evidence base for this treatment is very weak. Thus, the foundation of giving a recommendation of LT in clinical routine care is virtually non-existent. To further evaluate the benefit of LT in stage IV pan-NET, a prospective randomized trial is needed, however, very difficult to perform with the very limited number of eligible patients and relatively good prognosis in patients fulfilling current criteria.
